# Confocal-based fluorescence fluctuation spectroscopy with a SPAD array detector

**DOI:** 10.1038/s41377-021-00475-z

**Published:** 2021-02-05

**Authors:** Eli Slenders, Marco Castello, Mauro Buttafava, Federica Villa, Alberto Tosi, Luca Lanzanò, Sami Valtteri Koho, Giuseppe Vicidomini

**Affiliations:** 1 Molecular Microscopy and Spectroscopy, Istituto Italiano di Tecnologia, Genoa, Italy; 2 Dipartimento di Elettronica, Informazione e Bioingegneria, Politecnico di Milano, Milan, Italy; 3 Nanoscopy and NIC@IIT, Istituto Italiano di Tecnologia, Genoa, Italy; 4 Dipartimento di Fisica e Astronomia, Università di Catania, Catania, Italy

**Keywords:** Fluorescence spectroscopy, Microscopy

## Abstract

The combination of confocal laser-scanning microscopy (CLSM) and fluorescence fluctuation spectroscopy (FFS) is a powerful tool in studying fast, sub-resolution biomolecular processes in living cells. A detector array can further enhance CLSM-based FFS techniques, as it allows the simultaneous acquisition of several samples–essentially images—of the CLSM detection volume. However, the detector arrays that have previously been proposed for this purpose require tedious data corrections and preclude the combination of FFS with single-photon techniques, such as fluorescence lifetime imaging. Here, we solve these limitations by integrating a novel single-photon-avalanche-diode (SPAD) array detector in a CLSM system. We validate this new implementation on a series of FFS analyses: spot-variation fluorescence correlation spectroscopy, pair-correlation function analysis, and image-derived mean squared displacement analysis. We predict that the unique combination of spatial and temporal information provided by our detector will make the proposed architecture the method of choice for CLSM-based FFS.

## Introduction

Fluorescence fluctuation spectroscopy (FFS) is an ensemble of microscopy tools that allow biomolecular dynamics, interactions, and structural changes in living cells to be measured by studying temporal and/or spatial fluctuations in the fluorescence intensity^1^. Within the FFS family, fluorescence correlation spectroscopy (FCS)^2,3^ is the most popular technique. Indeed, FCS is readily available in all commercial confocal laser-scanning microscopes (CLSMs).

In CLSM-based FCS—also called single-point FCS—the excitation laser beam is focused by the objective lens, forming a diffraction-limited excitation volume at a static position in the sample. The light emitted by the fluorophores inside this excitation volume is collected by the same objective lens—filtered spectrally and spatially—and focused onto a single-point detector, such as a photomultiplier tube (PMT), an avalanche photodiode (APD), a single-photon avalanche diode (SPAD), or a hybrid detector (HyD). Dynamic processes in the sample, such as the diffusion of the fluorophores in and out of the detection volume (i.e., the excitation volume filtered by the pinhole), will lead to temporal fluctuations in the measured fluorescence signal intensity. These fluctuations are monitored for a relatively long period, typically from several seconds to minutes, with a microsecond sampling rate^2^. By calculating and analysing the (auto)correlation function of the intensity time trace, sub-resolution information about the underlying biomolecular processes causing these fluctuations can be revealed.

With the synergistic combination of CLSM with FCS, FFS techniques that include spatial information in a fluctuation analysis could be developed. In scanning-FCS^4–7^, the detection volume is scanned repetitively along a line or a circle—producing an intensity trace as a function of time and space, known as an intensity carpet. The temporal correlation curve is calculated for each position of the detection volume, and thus, many regions are probed almost in parallel—depending on the line/circle repetition rate. By using the same intensity carpet, in pair-correlation function (pCF) analysis^8^, pairs of intensity traces are temporally correlated to detect diffusion barriers and biomolecular connectivity. In raster image correlation spectroscopy^9^, spatio-temporal image correlation spectroscopy (STICS)^10^, image-derived mean squared displacement (iMSD) analysis^11^, and 2D-pCF analysis^12^, full 2D raster images are collected, and correlations are calculated in both time and space. These spatio-temporal correlation functions contain information on the speed and direction of active transport and the diffusion modality, i.e., free diffusion, confinement, partial confinement or dynamic partitioning. In spot-variation FCS^13^, several FCS measurements are performed with different detection volume sizes. The corresponding correlation functions are compared—similar to iMSD analysis—to assess the molecular environment organisation or to distinguish between different molecular diffusion modes.

Because of the various manners in which the image is formed and because of the different analysis methods, every FFS technique described above has its own characteristics in terms of spatial resolution, temporal resolution, and information content. Consequently, several separate correlative FFS experiments, usually on different microscopy systems, are required. Very recently, owing to the introduction of the AiryScan detector (in a nutshell, a core with a hexagonal-shaped 32-fibre bundle connected to a linear GaAsP-PMT detector array^14^), this complexity has been significantly reduced. AiryScan allows fast imaging of the fluorescence signal within a detection volume (up to 1.28 µs per image) instead of integrating all the signals, as was done with traditional detectors. Consequently, correlations in time and space between signals simultaneously collected from different parts of the detection volume can be analysed. In short, this technique, called comprehensive correlation analysis (CCA)^15^, combines the different FFS techniques in a single experiment with a broad temporal range (from microseconds to seconds), with a spatial resolution based on the size of the detection volume, and which is typically diffraction-limited.

However, in stark contrast to digital detectors, the AiryScan analogue detector precludes the implementation of other important advancements of FCS, such as time-resolved stimulated emission-depletion FCS (STED-FCS) for subdiffraction spot-variation FCS^16–18^, fluorescence lifetime correlation spectroscopy (FLCS)^19^ or pulse interleaved excitation FCS (PIE-FCS) for multicolour FCS^20^ and Förster resonance energy-transfer FCS (FRET-FCS)^21^. Although the temporal resolution in GHz of the analogue detectors is sufficient to measure photon arrival times, signal digitisation causes a photon-timing jitter, which is typically worse than that observed in digital detectors. Moreover, PMTs have been largely avoided in FFS analyses because the digitisation of the signal can introduce unwanted correlations. Finally, in the case of the AiryScan detector, the synchronous readout implementation completely precludes access to the photon arrival times.

**Fig. 1:**
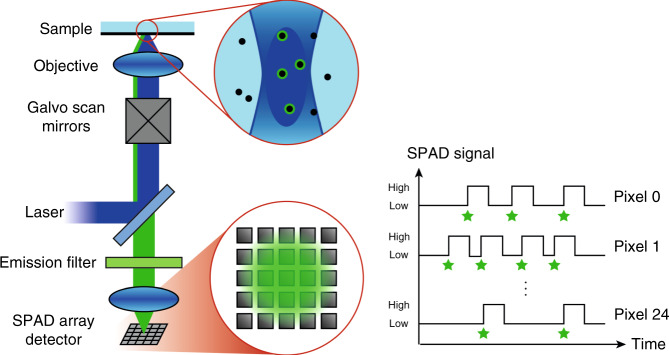
Imaging the fluorescence signal in a confocal setup with a SPAD array detector. The 25 pixels of the detector are numbered from 0 for the pixel in the top left corner to 24 for the pixel in the bottom right corner. Each photon, represented by a green star, instantaneously creates a local high-voltage signal in one of the SPAD pixels, which can be read out asynchronously and independently from the other pixels

Recently, our group introduced a new class of asynchronous-readout silicon SPAD array detectors^22^ with temporal and spatial characteristics that can overcome these limitations. Similar to the AiryScan detector, these SPAD array detectors allow imaging of the fluorescence detection volume but with a practically unlimited frame rate, and they temporally tag the fluorescence photons with picosecond precision; see Fig. 1. Indeed, upon the detection of a photon, each element fires a fast digital signal independently from the other elements. Due to the asynchronous readout, the frame rate is only limited by the nanosecond range hold-off (or dead-time) of each element^23^.

In this work, we show how this new class of SPAD array detectors allows for straightforward implementations of CCA and removes the limitations of an analogue (synchronous) readout detector array. In contrast to analogue detectors, the CCA implementation based on SPAD array detectors is correction-free, provides a sub-microsecond temporal resolution and is compatible with the many combinations of fluorescence lifetime measurements and FFS. For the sake of clarity, we want to emphasise that SPAD array detectors have already been used for FCS but only to parallelise single-point FCS^24–28^ or within a wide-field microscopy architecture^29^.

The most important advantage of our implementation is that the only technical differences with respect to a conventional (non-multifocal) CLSM-based FCS experiment are the detector and the data-acquisition (DAQ) system. Thus, our CCA implementation does not require any modification of the sample preparation protocol, complex data correction, or the acquisition of new experimental skills by the user. Basically, a single measurement provides the information that previously required several experiments—often on different microscopy systems—or that was lost during the fluorescence signal recording process. Therefore, we expect that SPAD-based CCA implementation will help answer many fundamental biomolecular questions, e.g., how nuclear organisation regulates nuclear trafficking to maintain genome function or how synaptic proteins are involved in synaptic signal transduction.

## Results

**Fig. 2:**
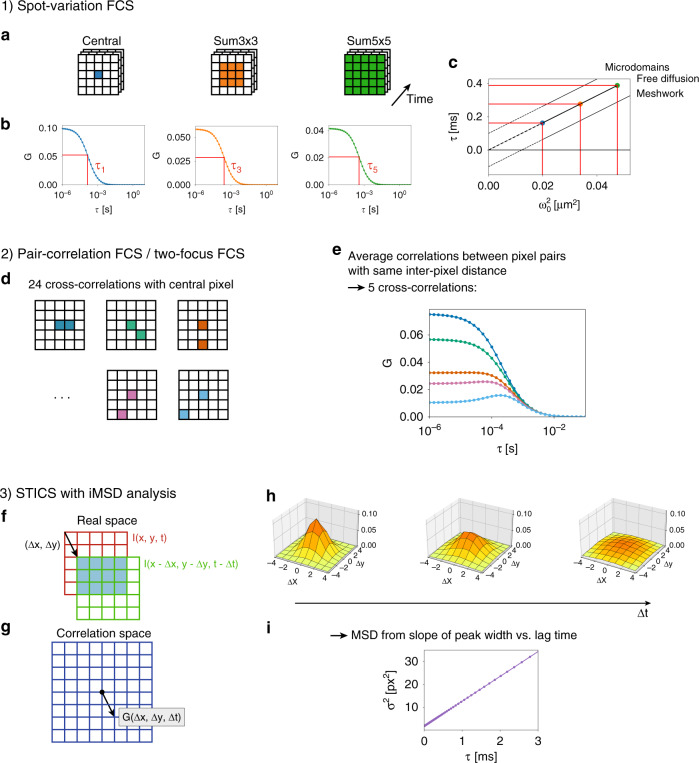
Overview of the analysis techniques (1) spot-variation FCS, (2) pair-correlation analysis, and (3) spatio-temporal image correlation spectroscopy (STICS) with image-derived mean squared displacement (iMSD) analysis. In spot-variation FCS, **a** the signal from different pixels is summed for each frame, **b** the correlation curves of the corresponding time traces are calculated and fitted, and **c** the resulting diffusion times τ are plotted as a function of the beam waist *ω*_0_^2^. Free diffusion yields a directly proportional relationship between *τ* and *ω*_0_^2^. Microdomains in the sample yield a positive intercept, *τ*(0) > 0, diffusion through a meshwork yields a negative intercept, *τ*(0) < 0. In two-focus FCS with pair-correlation analysis, **d** all 24 cross-correlations between the central pixel and the other pixels are calculated. **e** Cross-correlations between pixel pairs with the same interpixel distance are averaged, and the resulting 5 curves are simultaneously fitted in a global fit. **f** For STICS with iMSD analysis, each 5 × 5 frame is treated as an image. **g**, **h** The images are correlated in space and time, resulting in a series of 9 × 9 matrices. For free diffusion, the surface plots of these matrices show a Gaussian function of which the amplitude decreases and the width (*σ*^2^) increases linearly for increasing lag times. **i** The diffusion coefficient can be derived from the slope of the curve *σ*^2^(*τ*)

Each CLSM-based FFS measurement implemented with our SPAD array detector results in a three-dimensional (*x*, *y*, *t*) intensity dataset, which can be analysed in a variety of ways. Here, we propose three different ways to process a data set: (i) spot-variation FCS, (ii) pair-correlation analysis or two-focus FCS, and (iii) STICS with image-derived mean squared displacement analysis. The methods are schematically depicted in Fig. 2. Although the asynchronous-readout nature of our SPAD array detector theoretically provides an “unlimited” frame rate, in all our experiments, the data-acquisition system temporally integrated photon arrivals in bins of 500 ns. This bin width is compatible with most biomolecular processes.

**Fig. 3:**
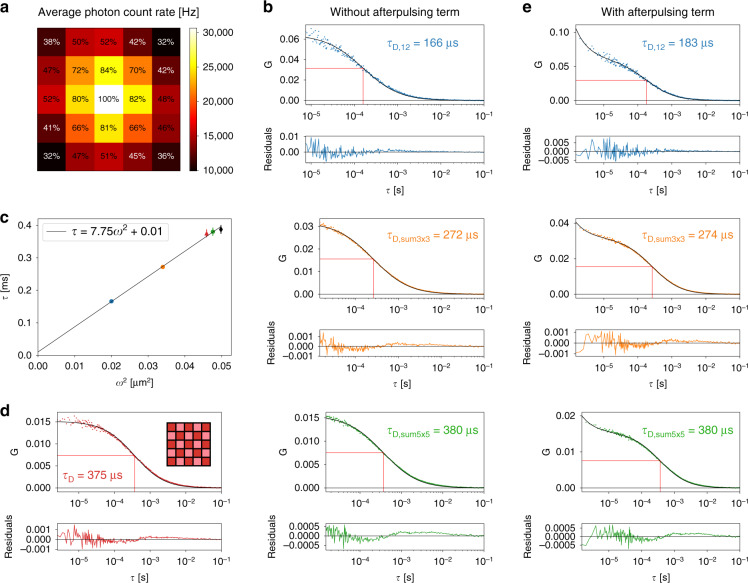
FCS measurements on Alexa 488 coupled to an antibody. **a** Average PCR for each pixel of the SPAD array detector. The percentages indicate the relative photon flux with respect to the central pixel. Pixel 1 is known as a hot pixel (see the SI for more details) and was not included in the analysis unless mentioned otherwise. **b** ACFs from *I*_12_ (cropped at 7.5 µs) and *I*_sum3×3_ and *I*_sum5×5_ (both cropped at 15 µs) in blue, orange, and green, respectively. **c** Diffusion time as a function of the square of the lateral PSF waist for the three configurations (same colour code as panel **b**). The error bars are standard deviations over four measurements. For *I*_12_ and *I*_sum3×3_, the error bars are too small to be visible. The data point represented by the black diamond shows, for comparison reasons, the results of I_sum5x5_ with all pixels included. The red triangle data point is derived from a chequerboard pattern cross-correlation analysis; see panel **d**. Line plot: linear fit of the three data points *I*_12_, *I*_sum3×3_, and I_sum5×5_. **d** Example of a chequerboard pattern cross-correlation. The data were cropped at *τ* = 2.5 µs. **e** Same ACFs as in **b** but showing the after-pulse component at short lag times. A power law factor A. *τ*^B^ with *B* = 1.103 and A as a fit parameter was included in the model to account for detector after-pulsing. The *τ*_D_ values in all panels were averaged over four FCS measurements of 200 s each

Figure 3a shows the average photon count rate (PCR) per pixel for one of the FFS measurements on Alexa 488 antibody-conjugate probes with an average laser power of 7 µW and a laser repetition rate of 80 MHz. We call this intensity distribution image the fingerprint map, and it represents the convolution of the excitation and emission point-spread-function (PSF) of the CLSM^23^. Future studies will focus on understanding whether the fingerprint map can be used to estimate both the excitation and emission PSF of the CLSM, thus avoiding the typical FFS calibration measurements required to obtain such system information. In this work, we show the fingerprint map, in particular its Gaussian-like shape, to demonstrate the ability of the SPAD array detector to “indirectly” image the fluorescence detection volume (or effective PSF). A direct measurement of the PSF would require a centred and immobilised probe.

### Spot-variation FCS

For spot-variation FCS, the spatial integration over the nine most central elements (*I*_sum3×3_(*t*)) and the integration over all pixels (*I*_sum5×5_(*t*)) were calculated for each temporal bin. Then, the autocorrelation of the central element time trace (*I*_12_(*t*)) and *I*_sum3×3_(*t*) and *I*_sum5×5_(*t*) were calculated. The resulting correlation curves, *G*_12_(*τ*), *G*_sum3×3_(*τ*), and *G*_sum5×5_(*τ*), showed a strong peak at short lag times caused by detector after-pulsing. To remove this component, the curves were cropped at short lag times before fitting the data. The theoretical equation for free diffusion in a 3D Gaussian volume model was used as a fit model^30^, but any other model can be implemented. From the fitted diffusion time *τ*_D_, one can derive either the lateral waist of the Gaussian PSF *ω*_0_ or the diffusion coefficient *D*. For free diffusion, *τ*_D_ is directly proportional to *ω*_0_^2^: *τ*_D_ = *ω*_0_^2^/4*D*. Here, we performed FCS on green fluorescent proteins (GFP) to calibrate *ω*_0_ for the three configurations, and then used these values to obtain the diffusion coefficient of Alexa 488-conjugated antibody probes.

An example of the ACFs for the Alexa 488 sample for the different detection schemes is shown in Fig. 3b. Averaging over different measurements (*N* = 4) yielded *τ*_D,12_ = (166 ± 7) µs, *τ*_D,sum3×3_ = (272 ± 7) µs, and *τ*_D,sum5×5_ = (38 ± 2) × 10 µs. For free diffusion, the average time a molecule spent in the focal volume is proportional to *ω*_0_^2^. Figure 3c shows a plot of *τ* as a function of *ω*_0_^2^ for the three configurations *I*_12_, *I*_sum3×3_, and *I*_sum5×5_. The linear fit had an intercept value *τ*(0) close to zero, confirming the absence of anomalous diffusion. Indeed, very similar values for the diffusion coefficients were found for the three data points: *D*_12_ = (30 ± 2) µm^2^ s^−1^, *D*_sum3×3_ = (31.3 ± 0.8) µm^2^ s^−1^, and *D*_sum5×5_ = (31 ± 2) µm^2^ s^−1^, which were close to the literature value of 30.6 µm^2^ s^−1^ ^31^.

Instead of cropping the ACFs to remove the after-pulsing component, an additional power law component *A* × *τ*^B^ could be added to the fit model^26^. This component contains two additional parameters, A and B, the latter of which was measured by fitting the autocorrelation curve of the central element in a reference measurement under weak ambient light; see Fig. S2c. Notably, this value is a property of the SPAD detector and does not depend on the experiment, which means that it needs to be measured only once. Similar values could be derived for the other elements. Given parameter B, parameter A was kept free in the FCS fit. Figure 3e shows an example. The diffusion times averaged over multiple measurements (*N* = 4) were (183 ± 3) µs, (274 ± 7) µs, and (38 ± 2) × 10 µs, in good agreement with the values obtained by cropping the autocorrelation curve. The corresponding diffusion coefficients were (30.4 ± 0.5) µm^2^ s^−1^, (32.6 ± 0.8) µm^2^ s^−1^, and (32 ± 2) µm^2^ s^−1^.

A third way of eliminating the effect of after-pulsing was to calculate cross-correlations between the signals from different detector elements, as their after-pulsing signals did not correlate. As an example, Fig. 3d shows the cross-correlation between the sum of all the odd-numbered elements and the sum of all the even-numbered elements, i.e., a chequerboard pattern cross-correlation. By grouping the 25 channels into two photon streams in a chequerboard pattern, two independent intensity time traces were obtained. Both traces were recorded with a very similarly sized detection volume, and both detection volumes were centred at the same position. Consequently, the conventional analytical model for FCS in three dimensions could be used to fit the data. In this case, the absence of the after-pulsing component also allowed data points at short lag times to be included. The resulting diffusion coefficient was (31 ± 1) µm^2^ s^−1^, and the corresponding data point in Fig. 3c was close to the fitted curve.

### Two-focus FCS

The geometry of the SPAD array detector makes two-focus FCS^32^ or, equivalently, pair-correlation FCS straightforward. The field of view of every pixel was laterally shifted with respect to the field of view of the central pixel. Cross-correlating the signals from different pixels is therefore equivalent to two-focus FCS. The intensity trace from the central pixel *I*_12_(t) was taken as the reference signal, and the 24 cross-correlations with the traces from the other pixels were calculated. In the absence of after-pulsing, the autocorrelation of *I*_12_ could also be included. We used the notation *G*_i,j_ to denote the cross-correlation between pixels *i* and *j*. All cross-correlation curves corresponding to equal interpixel distances were averaged, resulting in five cross-correlation curves. For example, pixels 2, 10, 14, and 22 were all two units away from the centre of the detector (i.e., *ρ* = 2). Thus, *G*_12,2_, *G*_12,10_, *G*_12,14_, and *G*_12,22_ were averaged. The five curves were simultaneously fitted with the two-focus FCS model, assuming equal-sized Gaussian detection volumes for each pixel^33^.

**Fig. 4:**
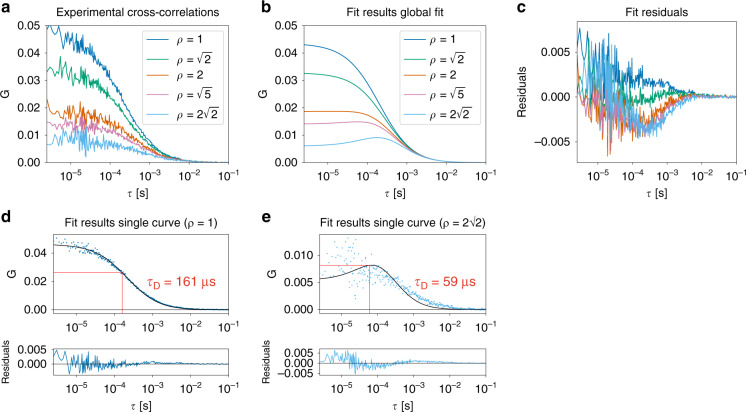
Pair-correlation analysis on the Alexa 488 sample. **a** Experimental cross-correlation functions between the central pixel and the other pixels, **b** global fit, and **c** fit residuals for one of the measurements. The fit result (*N* = 4) is *D* = (32.5 ± 0.6) µm^2^ s^−1^. The PSF size was kept fixed at *ω*_0_ = 141 nm, i.e., the value that was found with the GFP reference sample. The parameter *ρ* represents the distance between the two pixels of which the signals are correlated: *ρ* = 1 corresponds to the distance between the central pixel and a laterally neighbouring pixel, *ρ* = $\sqrt 2$ corresponds to the distance between the central pixel and a diagonally neighbouring pixel, etc. A distance of *ρ* = 1 corresponds to a shift in the field of view of 75 nm. For **c**, the same colour code was applied as for the other panels. **d**, **e** Experimental cross-correlation functions and individual fits for **d** *ρ* = 1 and **e** *ρ* = $2\sqrt 2$. The *τ*_D_ values in red represent the diffusion times obtained from the fitted curves in black

Figure 4 shows an example of the cross-correlations between the central pixel and the other pixels for the Alexa 488 sample. The distance between the different focal volumes was fixed at the theoretical values (Supplementary Note 4).

The global fit, as shown in Fig. 4b, results in a single value for the diffusion coefficient of *D* = (32.5 ± 0.6) µm^2^ s^−1^ (*N* = 4). The fitted value was close to the expected value but was less accurate than the results obtained with spot-variation FCS and fluorescence cross-correlation spectroscopy. The fit residuals, panel (c), were not completely random. The main reason for the poorer performance of this analysis method was the contribution of the cross-correlations with high *ρ* values. When each curve was fitted separately (Fig. 4d, e), the diffusion coefficient for *ρ* = 1 was (30.6 ± 0.7) µm^2^ s^−1^, whereas *ρ* = $2\sqrt 2$ yielded *D* = (88 ± 4) µm^2^ s^−1^ (*N* = 4). Clearly, cross-correlating data from pixels that were far away from each other reduced the accuracy. This effect was to be expected, given that the amplitude of the correlation function, and hence the signal-to-noise ratio, decreases with the square of the lateral shift. Moreover, pixels far away from the optical axis had a detection volume that deviated significantly from a Gaussian function; hence, a different fit model would be useful to analyse these cross-correlations. Alternatively, one could increase the magnification on the detection side. For example, if the detector size was set to 1 A.U. instead of 1.5 A.U., the detection volumes for the pixels near the edge of the detector would more closely resemble a Gaussian function but at the cost of a lower signal-to-noise ratio (SNR).

Although pair-correlation analysis is less accurate when pixels far away from one another are correlated, the technique has a significant advantage over spot-variation FCS in the case of anomalous diffusion. In spot-variation FCS, the signals of different pixels are summed, depending solely on their distance with respect to the optical axis. Consequently, all information on directionality is lost. In contrast, in pair-correlation analysis, the cross-correlation function of the signals of two (neighbouring) pixels reveals information about the anisotropy of the sample. The technique is particularly suited to measuring active transport since the cross-correlation function yields information on the speed of the transport in the direction of the line connecting the two pixels.

### Intensity mean squared displacement

Instead of viewing the measurement data as 25 *I*_x,y_(*t*) individual time traces, one could also consider the data as a time series of images. Correlations could then be calculated in space and time, similar to STICS analysis^10^. First, the cross-correlations between all possible combinations of pixel pairs were calculated. Then, all pair correlations corresponding to equal spatial shifts were averaged, leading to a 9 × 9 correlation matrix for each lag time. Every correlation matrix was fitted with a 2D Gaussian function, as was done in iMSD analysis, and the variance *σ*^2^ was plotted as a function of *τ*. The resulting curve was fitted with a first-order polynomial function from which the diffusion coefficient was obtained: *σ*^2^ = 2*Dτ* + *ω*_0_^2^/2.

**Fig. 5:**
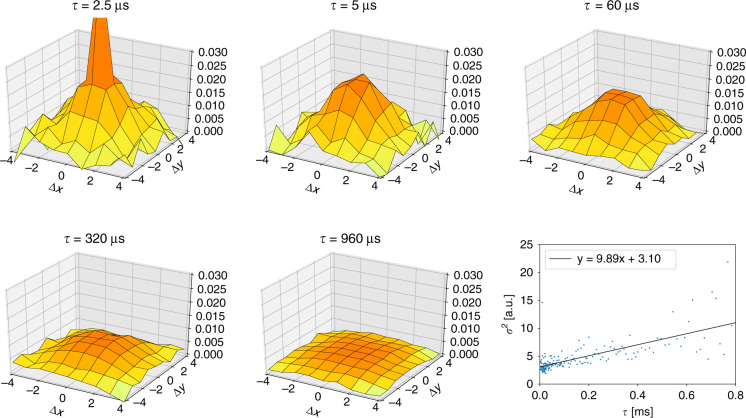
STICS analysis on the Alexa 488 sample for different *τ* values. The peak at (0, 0) for *τ* = 2.5 µs is the consequence of detector after-pulsing. This peak was removed in the other panels for visualisation purposes. The line plot shows the variance of the 2D Gaussian fits *σ*^2^ (expressed in terms of pixels on the SPAD detector) as a function of *τ*. The diffusion coefficient calculated from the slope is 27.8 µm^2^ s^−1^

Figure 5 shows the results of a STICS correlation analysis. As predicted by the model, a 2D Gaussian-like function was observed in which the amplitude decreased and the variance increased for increasing lag times. The sharp peak at (0, 0) for small lag times was caused by detector after-pulsing and was not included in the fit analysis. For free diffusion, the centre of the Gaussian distribution remained at (0, 0), and the variance *σ*^2^ increased linearly with increasing lag times. The slope of the curve was equal to 2D, independent of the detection volume size. The STICS analysis was therefore a calibration-free method. Figure 5 shows the fitted variance as a function of the lag time. The SNR of the correlation surface decreased with increasing lag times as the amplitude went to zero. Therefore, we only considered lag times below 800 µs. From the slope of the fit, which was equal to twice the diffusion coefficient, a value of *D* = 28 µm^2^ s^−1^ was found. The iMSD method is therefore less accurate than the other methods, but it has the advantage of being calibration-free. The diffusion coefficient can be derived from the slope of *σ*^2^(*τ*), the geometry of the detector, and the magnification of the system, all of which are well-known quantities.

## Discussion

In this paper, we demonstrated the use of a new class of asynchronous-readout SPAD array detectors for CCA in a confocal laser-scanning microscope. In particular, we showed that by employing an array detector, the implementation of several FFS techniques, such as conventional single-spot FCS, spot-variation FCS, 2D-pCF, and iMSD analysis, is extremely straightforward in a single experiment. No data corrections or additional calibration measurements, except those typical of FFS, had to be introduced. Table S1 provides an overview of these analysis methods presented here and their advantages and limitations. We applied and validated our techniques to freely diffusing GFP and an antibody coupled to Alexa 488, demonstrating the potential application of SPAD-based CCA to study more realistic anomalous biomolecular diffusion dynamics in living cells.

The current generation of 0.35 µm high-voltage complementary metal-oxide-semiconductor (HVCMOS) SPAD detectors suffers from after-pulsing, but we have shown several methods to compensate for this effect. It is worth noting that other types of SPAD technology fabrication, such as 0.16 µm bipolar-CMOS-DMOS (BCD)^22^, have an after-pulsing probability far below 1% at the same hold-off time—more than one order of magnitude less than the HVCMOS technology used here. On the other hand, the dark count rate of BCD SPAD array detectors can be significantly higher than that of their HVCMOS counterparts, but since the dark counts were uncorrelated, they would not severely affect the FCS autocorrelation curves. In addition, cooling the detector array could significantly reduce the DCR. The potential of BCD technology for FCS experiments is demonstrated in the Supplementary Information, Figure S3, which shows an example of an FCS experiment using the Alexa 488 sample. Fitting these data with the conventional FCS model and without cropping the data yielded diffusion times that were very similar to the values presented above.

The hold-off time of single-element SPAD detectors severely limits the use of such a detector in high photon count rate FCS, e.g., in samples with high concentrations of bright fluorophores^34^. However, in a SPAD array detector, each pixel operates as an independent detector. Since the probability of two consecutive emission photons hitting the same pixel is small, as the flux of the emission photons is spread over the array following the emission point-spread function, the maximum photon count rate is significantly increased. The parallelisation of the array detector thus allows the arrival times of multiple photons generated in a single excitation pulse to be measured. Notably, this unique ability of the SPAD array detector can open up new exciting combinations of CCA and photon-correlation analysis^35^.

One of the most important benefits of the SPAD array detectors used in this work, precluded by the AiryScan detector, is their compatibility with photon time-tagging. For this reason, the next natural step will be to combine CCA with fluorescence lifetime analysis. To this end, the digital signals generated by the detector, which have a temporal resolution on the order of picoseconds, need to be properly acquired and transferred to a computer. Connecting the detector to a multichannel time-tagging data-acquisition platform allows the arrival time of each photon to be tagged with a precision on the order of 100 ps with respect to the sync signal of a pulsed laser. It is therefore possible to reconstruct the fluorescence decay histogram for each element of the array.

It should be noted that several groups recently demonstrated measuring fluorescence lifetimes with two-dimensional SPAD array detectors with a high number of pixels^36^, mainly in combination with wide-field microscopy or with a multibeam scanning microscopy architecture. Large SPAD array detectors typically require internal (in-pixel) processing of the lifetime information by implementing time-gating^37–39^ or by in-pixel calculations of the fluorescence histogram^40^. These techniques reduce data transfer, but potentially crucial arrival time information is lost. Consequently, these lifetime measurements cannot readily be combined with the FFS techniques described above. In contrast, our detector has a relatively small number of pixels (i.e., 5 × 5 instead of 32 × 32 or higher), which means that transferring time-tagging data for each photon is feasible. The small number of pixels of our detector limits its applications in the context of wide-field imaging or multibeam CLSM, but in the context of single-beam CLSM-based FFS, which is the topic of this work, our SPAD array detector offers the best compromise between spatial sampling of the detection volume and performance. Indeed, although a higher number of pixels (e.g., 7 × 7) will, for example, result in more data points for spot-variation FCS analysis, there is a cost: the greater amount of data transfer increases the system complexity, and the specifications, such as the overall dark noise, can worsen. We strongly believe that our recently developed small pixel-number SPAD array, designed to sample the CLSM detection volume, has been a game changer. This statement is supported by the fact that different companies and research groups specialising in photodetectors have released similar small single-photon detector arrays.

Together with after-pulsing, dark noise, hold-off time, and photon-timing precision, other SPAD array detector characteristics, such as photon-detection efficiency, fill factor and cross-talk, are important to obtain robust and sound SPAD-based CCA measurements. Since these characteristics are strictly connected, their simultaneous optimisation is not straightforward and may require a synergistic combination of different approaches, such as new SPAD array fabrication technologies and the introduction of micro-lenses, optical trenches, and cooling systems.

The time-tagging information available in fluorescence lifetime correlation spectroscopy (FLCS) offers a series of advantages over conventional FCS. First, the molecular environment or conformational state of a biomolecule is often linked to the fluorescence lifetime. FLCS thus allows the simultaneous characterisation of the structure or environment of a biomolecule, its dynamics, and the potential correlation between the two. Second, arrival time information can be employed to separate auto-correlations or cross-correlations of different fluorescent species that emit in the same spectral range by filtering the data based on the individual fluorescence decay functions^41^. Alternatively, interactions between different biomolecular species can be analysed through multicolour CCA via pulsed-interleaved excitation, which can be used to efficiently integrate FRET analysis. Third, FLCS also enables the correction of the after-pulsing problem and background in addition to bleedthrough removal, thus producing more accurate values for the concentrations and diffusion coefficients.

One problem of spot-variation FCS is that it provides an indirect analysis of the different diffusion modalities because the detection volume can only be reduced down to the limit imposed by the diffraction of the light. The combination of spot variation with STED microscopy has solved this problem, since different subdiffraction detection volumes can be obtained by increasing the intensity of the STED beam (STED-FCS)^13^ or, more importantly, in this context, by analysing how the fluorescence decay histogram is perturbed by the STED beam^16–18^. A new class of FFS techniques can be implemented not only by synergistically combining time-resolved STED-FCS with the spot-variation approach described above, but also by combining time-resolved STED-FCS with CCA. The feasibility and importance of this combination is also demonstrated by the recent integration of our SPAD array detector into our STED microscope^42^.

Fast (much faster than the typical pixel dwell time of a CLSM) detector arrays, such as AiryScan and our asynchronous readout SPAD array, are revolutionising imaging in laser-scanning microscopy^23,43^. These detectors can effectively transform any LSM into a super-resolution microscope without sacrificing any characteristics of this well-established microscopy technique. Furthermore, our SPAD array detector provides access to fluorescence lifetime information. By demonstrating that the SPAD array detector can also significantly boost FFS analyses, we expect that this work can further speed up a scenario in which single-element detectors disappear from laser-scanning microscopes and are substituted by a SPAD array detector. This scenario is also supported by the considerable momentum in the development of SPAD array detectors^36^.

## Materials and methods

### Microscope and data-acquisition systems

Our SPAD-FFS system was built as a modification to a confocal laser-scanning microscope and has the same architecture as an image-scanning microscope^23^. A 485 nm 80 MHz pulsed laser (LDH-D-C-485 PicoQuant, Berlin, Germany, driven by a PicoQuant PDL 828 Sepia II Multichannel Picosecond Diode Laser driver) was used for excitation. After passing through a 488/10 nm clean-up filter, the laser light was reflected by a dichroic beam splitter (ZT405/488/561/640 rpc, Chroma Technology Corporation, Vermont, USA) towards the galvanometric scanning mirrors). The scanning system was coupled to a 50 mm Leica scan lens and a 200 mm Leica tube lens. All measurements were performed with a 100×/1.4 Leica objective. Axial scanning, essential for measuring the structural parameter of the PSF, was implemented with a piezo stage (Nanopositioning system, Mad City Labs Inc., Madison, USA). The fluorescence signal was collected in de-scanned mode, in which it passed through a dichroic beam splitter, a 488 nm notch filter and a fluorescence emission filter (ET570/60, Chroma Technology Corporation). A 250 mm lens conjugated with the scan lens was installed to obtain a 1.5 Airy unit field of view on the SPAD array detector, which meant that the detector also acted as a pinhole to remove the out-of-focus fluorescence background.

The measurements were performed with a 5×5 silicon SPAD array detector fabricated using 0.35 µm HVCMOS technology^22^. Compared to other larger SPAD detectors^37,39^, this detector has a much higher fill factor (approximately 50%) and is therefore better suited for imaging the detection volume. The hold-off time ranged from 25 to 500 ns and could be chosen by the user (the longer the hold-off, the lower the after-pulsing probability, and the lower the maximum count rate). The photon time-jitter of the output pulses was ∼150 ps, which made the detector perfectly suited for fluorescence lifetime experiments^23^. More details on the specifications of the detector can be found in Supplementary Note 2. The detector was connected to a multifunction data-acquisition card (USB-7856R, National Instruments, Texas, USA) that was driven by a custom-built LabVIEW FPGA programme. On the FPGA level, the cumulative number of photons detected in each pixel since the start of the measurement was stored and updated at a rate of 200 MHz. Every 100 cycles, i.e., every 0.5 µs, the absolute number of photons detected during the last 100 cycles was calculated and sent to a PC, where a high-level LabVIEW programme collected, plotted and stored the data. The detector allows asynchronous readout, but a bin time of 0.5 µs was sufficient for the FCS experiments described here. The dataset obtained for each measurement was no longer a single intensity time trace (as for conventional FCS) but 25 intensity time traces (one per element) or, equivalently, an intensity time series of 5 × 5 images. To compress the data and limit the bit rate, the absolute photon counts for all pixels in a single bin were converted into a 128-bit string before being sent to the PC. More bits were allocated for pixels closer to the centre of the detector, since these pixels were more likely to collect a high number of photons. This way, the bit rate could be limited to 30.5 MB s^−1^, which could be transferred via USB-2 and stored in real-time as a binary data file on a PC (Precision tower 5810, Dell Inc., Texas, USA). In addition, a text file with the measurement metadata was automatically generated by LabVIEW.

Several SPAD-FFS measurements that were several tens of seconds each were performed on two samples: GFP and Alexa 488 coupled to an antibody, both freely diffusing in water. The GFP data sets were used to calibrate the different detection volume sizes used successively for most of the FFS analyses of Alexa 488. For example, volume calibration is needed to convert Alexa 488 diffusion/transit times to absolute diffusion coefficients. All data were analysed in Python. The three different ways that we proposed to process a SPAD-FFS dataset (spot-variation FCS, pair-correlation FCS, and STICS with iMSD analysis) are schematically depicted in Fig. 2. The corresponding analytical fit functions are described in Supplementary Note 1. All our Python code and an exemplary data set are available on GitHub (https://github.com/VicidominiLab/spad-ffs).

### Calculation and fit of the correlation curves

The time traces for the different configurations (such as *I*_12_(*t*), *I*_sum3×3_(*t*), *I*_sum5×5_(*t*), etc.) were split into traces of 10 s, the auto-correlation or cross-correlation of each trace was calculated using the Multipletau Python package^44^, and then all curves were averaged. Correlations were calculated for logarithmically scaled lag times ranging between 500 ns and 0.1 s. The least squares optimiser of the SciPy library was used to fit the correlation curves.

### Sample preparation

#### GFP

To prevent GFP from sticking to the cover slip, the cover slip surface was coated with bovine serum albumin (BSA). One milligram of BSA was diluted in 100 µL of phosphate-buffered saline (PBS) and poured onto a cover slip. After 1 h, the solution was removed, and the cover slip was washed twice with PBS. Affinity-purified recombinant Aequorea coerulescens green fluorescent protein (rAcGFP1, Cat. No. 632502, Takara Bio Inc., Shiga, Japan) was diluted 200× in PBS, resulting in a final concentration of 185 nM, and 100 µl of the sample was poured onto the cover slip.

#### Alexa 488 AB

A goat antimouse antibody coupled with Alexa 488 (Ref. A11029, ThermoFisher) was diluted 100× in PBS, resulting in a final concentration of 20 µg mL^−1^. The suspension was sonicated for 10 min in a water bath sonicator (Labsonic LBS1–0.6, FALC Instruments, Treviglio, Italy), and 100 µL was poured onto a cover slip.

### Measurement of the detection volumes with GFP

To extract the diffusion coefficient from an FCS measurement, the beam waist *ω*_0_, i.e., the 1/*e*^2^ radius of the Gaussian detection volume (or PSF), must be known. The beam waist can be measured by performing FCS on a reference sample with a known diffusion coefficient. Here, we used GFP to measure *ω*_0_ for the different detection schemes, i.e., for *I*_12_, *I*_sum3×3_, and *I*_sum5×5_.

**Fig. 6:**
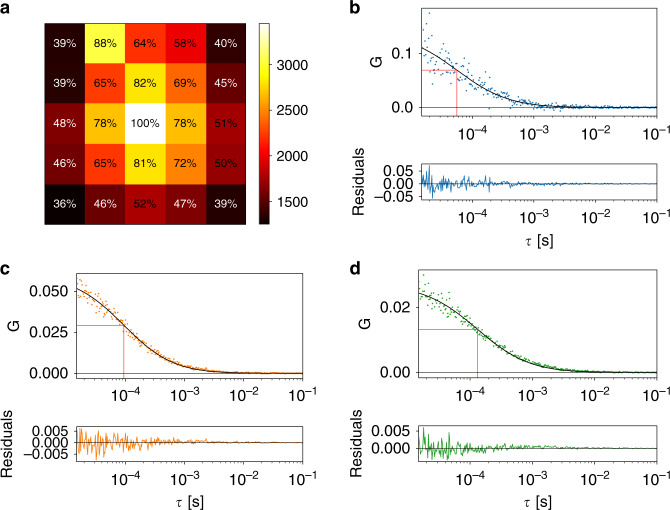
FCS measurements on GFP. **a** Average PCR (in Hz) per pixel of the SPAD array detector for one of the FCS measurements. The percentages indicate the relative photon flux with respect to the central pixel. **b**–**d** ACFs and fits for **b** *I*_12_, **c** *I*_sum3×3_, and **d** *I*_sum5×5_. The fitted diffusion times indicated in red correspond to 57 µs, 94 µs, and 132 µs, respectively

Four measurements of at least 200 s each were performed with laser powers ranging from 7.0 to 9.6 µW; the four measurements consisted of three measurements of 200 s with the detector hold-off value set to 500 ns and one measurement of 500 s with a hold-off value of 150 ns. Figure 6a shows the average PCR per pixel for one of the measurements. Figure 6b–d shows a typical example of the ACFs. All curves were cropped at *τ* = 15 µs to remove the after-pulsing component. Then, the data were fitted with the amplitude and the diffusion time as fit parameters. The structural parameter *z*_0_/*ω*_0_, with *z*_0_ being the height of the focal volume, was kept constant at the values found by measuring the PSF in 3D with fixed fluorescent beads. The average and standard deviation over the four measurements were *τ*_D,12_ = (56 ± 5) µs, *τ*_D,sum3×3_ = (94 ± 2) µs, and *τ*_D,sum5×5_ = (132.3 ± 0.9) µs. The corresponding beam waists could be calculated as $\sqrt {4D\tau _{\rm{D}}}$, with *D* = 90 µm^2^ s^−1^ being the diffusion coefficient for GFP^18^. The results were *ω*_0,12_ = 141 nm, *ω*_0,sum3×3_ = 184 nm, and *ω*_0,sum5×5_ = 218 nm.

## Supplementary information

Supplemental Material

## Supplementary information

**Supplementary Information** The online version contains supplementary material available at 10.1038/s41377-021-00475-z.
