# Supplementary material for: Confocal-based fluorescence fluctuation spectroscopy with a SPAD array detector
Source: Light Sci Appl. 2021 Feb 5;10:31. doi: 10.1038/s41377-021-00475-z (PMC7862647; doi:10.1038/s41377-021-00475-z)
Supplement: Supplementary file 1 — Supplemental Material [file 41377_2021_475_MOESM1_ESM.pdf]

Supplementary information:

Confocal-based Fluorescence Fluctuation Spectroscopy with a SPAD Array Detector

Eli Slenders[1], Marco Castello[1], Mauro Buttafava[2], Federica Villa[2], Alberto Tosi[2], Luca Lanzanò[3,4], Sami Valtteri Koho[1], Giuseppe Vicidomini[1].

[1]Molecular Microscopy and Spectroscopy, Istituto Italiano di Tecnologia, 16152 Genoa, Italy;

[2]Dipartimento di Elettronica, Informazione e Bioingegneria, Politecnico di Milano, 20133 Milan, Italy;

[3]Nanoscopy and NIC@IIT, Istituto Italiano di Tecnologia, 16152 Genoa, Italy;

[4]Dipartimento di Fisica e Astronomia, Università di Catania, 95123 Catania, Italy;

Supplementary Note 1: Theory

Let ω_0 and ω_1 be the $1/e^2$ radii of two Gaussian detection volumes (often the detection volume is called point-spread-function, PSF, in the context of imaging, or collection-efficiency function, CEF, in the context of FCS) and ζ_1 and ζ_2 their respective $1/e^2$ heights. Let the spatial shifts between the centers of the two focii be given by the vector $\vec{\rho}$. Assuming free diffusion with diffusion coefficient D and fluorophore concentration $\langle c \rangle$, the pair-correlation between the fluorescence signals $I_1(t)$ and $I_2(t)$ detected by the two detectors having the respective properties described above, can be written as

$$G(\vec{\rho},\tau) = \frac{2\sqrt{2}}{\pi^{3/2}\langle c \rangle (8D\tau + \omega_0^2 + \omega_1^2)\sqrt{8D\tau + \zeta_0^2 + \zeta_1^2}} \cdot \exp\left(-\frac{16D\tau\vec{\rho}^2 + 2(\zeta_0^2 + \zeta_1^2)(\rho_x^2 + \rho_y^2) + 2(\omega_0^2 + \omega_1^2)\rho_z^2}{(8D\tau + \omega_0^2 + \omega_1^2)(8D\tau + \zeta_0^2 + \zeta_1^2)}\right). \tag{1}$$

Eq. 1 is the general correlation formula from which specific cases can be derived. For conventional FCS, or spot-variation FCS, only a single detector is assumed. Substituting $\vec{\rho} = 0$, $\omega_1 = \omega_0$, and $\zeta_1 = \zeta_0$, Eq. 1 simplifies to the conventional FCS formula:

$$G(\tau) = \frac{2\sqrt{2}}{\pi^{3/2}\langle c \rangle (8D\tau + 2\omega_0^2)\sqrt{8D\tau + 2\zeta_0^2}} = \frac{1}{\pi^{3/2}\langle c \rangle (4D\tau + \omega_0^2)\sqrt{4D\tau + \zeta_0^2}}. \tag{2}$$

For fluorescence cross-correlation spectroscopy and two-focus FCS, Eq. 1 can be simplified assuming both PSFs have the same focal plane (*i.e.,* $\rho_z = 0$):

$$G(\rho,\tau) = \frac{1}{N\left(1 + \frac{\tau}{\tau_D}\right)\sqrt{1 + \frac{4D\tau}{\tau_D SP^2}}} \exp\left(\frac{-\rho^2}{\left(1 + \frac{\tau}{\tau_D}\right)\omega_{\text{eff}}^2}\right), \tag{3}$$

where $N = \pi^{3/2}\omega_{\text{eff}}^2 z_{\text{eff}} c$ is a measure of the average number of particles in either focal volume, $\tau_D = \frac{\omega_{\text{eff}}^2}{4D}$ is the effective diffusion time, $\omega_{\text{eff}}^2 = \frac{\omega_0^2+\omega_1^2}{2}$ is the effective squared beam waist, $z_{\text{eff}}^2 = \frac{z_0^2+z_1^2}{2}$ is the effective squared beam height, $SP = \frac{z_0}{\omega_0} = \frac{z_1}{\omega_1}$ is the shape parameter of the PSFs (which are assumed to be equal), and ρ is the distance between the centers of the two PSFs.

For the STICS approach with iMSD analysis, we assumed ω_0 to be equal for all pixels. The exponential factor in Eq. 3 can be rewritten as:

$$G(\rho, \tau) \sim \exp\left(\frac{-\rho^2}{4D\tau + \omega_0^2}\right). \tag{4}$$

Fitting the correlations with a 2D Gaussian function, $y = \exp\left(-x^2/2\sigma^2\right)$, with σ^2 the variance results in the following relationship:

$$\sigma^2 = 2D\tau + \frac{\omega_0^2}{2}. \tag{5}$$

The diffusion coefficient can thus be extracted from the slope of the $\sigma^2(\tau)$ curve without needing information on the size of the PSF.

Supplementary Note 2: SPAD array detector properties

As SPAD technology is constantly improving, also the specifications of SPAD array detectors are continuously getting better. This section provides an overview of the key properties of the HVCMOS SPAD array detector used in this work. More details about the SPAD array detector used in this work can be found in [4].

Physical dimensions

A sketch of the layout of the detector used in this work is shown in Figure S1. With a pixel size

p_s, and pixel pitch p_p of $50\ \mu m$ and $75\ \mu m$, respectively, the detector has a fill factor $f = p_s^2/p_p^2$ of about 44%. However, it is important to note that in this application the photons are not uniformly spread across the sensitive area of the detector, but instead follow a distribution that depends on the PSF of the system (Fig. 3(a)). In particular, photons are concentrated in the central area, thus the effective fill-factor, *i.e.*, the number of photons effectively reaching the sensitive area of the detector, is higher than 44%. In future updates of this detector, the effective fill-factor will be further improved by introducing a set of micro-lenses in front of the SPAD array.

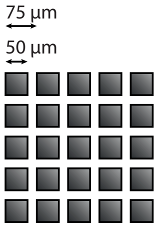

Figure S1. Sketch of the SPAD array detector. The size and pitch of the pixels are indicated.

Timing properties

Each pixel of the array operates as an independent SPAD. Upon detection of a photon, a logical high voltage pulse is almost instantaneously generated in that pixel. The photon-time jitter of the pulse is ~ 150 ps. The hold-off can be chosen by the operator and can range from 25 to 500 ns. During this hold-off time, no other photons can be detected by this pixel. Each pixel can be connected to a time-tagging platform for time-correlating single-photon counting experiments.

Dark photon count rate

Figure S2 (a) shows the number of photons detected by each pixel of the SPAD array detector in a dark environment. All pixels have a dark count rate (DCR) fluctuating between 85 and 200 Hz, except for one "hot" pixel, which has a DCR between 1.3 and 1.6 kHz. The hot pixel (pixel 1)

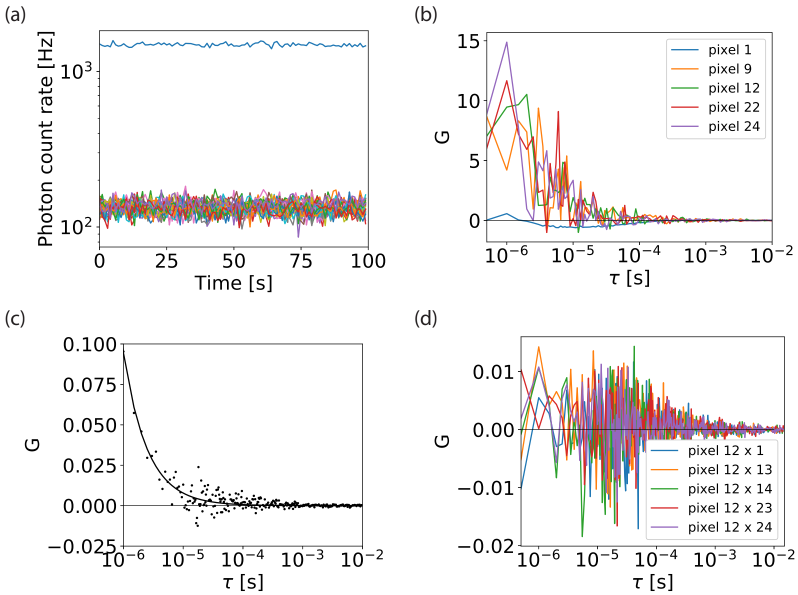

Figure S2. (a) Dark photon count rate (PCR) of the 25 pixels of the SPAD array detector. Measurement duration 100 s, data binned in bins of 1 s. The hold-off was set to 500 ns. All pixels have an average dark PCR between 100 and 150 Hz, except for pixel 1, which has an average dark PCR of more than 1000 Hz. (b) auto-correlation of a dark current measurement. For visualization purposes, only 5 of the 25 pixels are plotted. (c) auto-correlation of pixel 12 under weak ambient light. The data was fitted with a power law function $y = Ax^B$, with A and B the fit parameters. (d) Cross-correlations between the central pixel and five of the other pixels under weak ambient light. All panels show data recorded with a hold-off of 500 ns.

is located near the detector edge (*i.e.,* first row, second column) and therefore has only a minor influence on G_{sum5x5} and no influence at all on G_{sum3x3} and G_{12}. For this reason, we excluded the data collected by this pixel for the spot-variation measurements. It is important to note that for the CMOS SPAD the hot-pixel problem can be removed by a careful selection of the device. Alternatively, an active cooling system can be implemented, which, for silicon SPADs, guarantees approximately a decade in DCR decrease every 20 K of temperature reduction [41].

Afterpulsing

Figure S2 (b) shows the auto-correlation curves calculated for several pixels without illumination. Pixels are numbered starting from 0 for the pixel in the upper left corner up to 24 for the pixel in the lower right corner. Except for pixel 1, all auto-correlation curves follow the same pattern, starting at

a high value at short lag times and decreasing to 0 near 10 μs. This behavior is caused by detector afterpulsing, and, clearly, has to be taken into account in FCS measurements, as it will constitute a background in the auto-correlation curve.

There are various ways to correct for detector afterpulsing. The most straightforward method is cropping the FCS auto-correlation curve, thereby removing the spurious data points at short lag times. This strategy is justified in many biological applications, as the diffusion in biological samples is often relatively slow. However, when short lag time information is required, a different approach is needed. One method consists in characterizing the after-pulse component, e.g. by calculating the auto-correlation function (ACF) of the detector signal under constant white light illumination and fitting the data with a power law, as shown in Figure S2 (c). The same power law factor can then be added to the FCS fit model[5]. A second method is calculating FCS cross-correlations between the signal coming from different pixels. In this case, the afterpulsing effect can be completely filtered out, as the afterpulsing signals do not correlate, see Figure S2 (d). Moreover, the different spatial location of each pixel leads to a different position of the corresponding detection PSF in the sample plane. In other words, cross-correlating signals between different pixels is equivalent to performing two-focus FCS. In its original implementation[3], the shift between the PSFs was imposed on the excitation side by focusing two interleaved pulsed laser beams to a slightly different lateral position in the sample. Linking each photon to either excitation volume was done by means of a time-tagging platform. FCS based on SPAD array detection offers the same information with a much simpler experimental setup. In addition, the spatial shifts between the PSFs are fixed by the geometry of the detector, removing the need for a calibration measurement. A third method to remove the afterpulsing component at short lag times is via a combined FCS and fluorescence lifetime measurement[6]. The microsecond time scale of the afterpulsing is much longer than the nanosecond time scale between two excitation pulses. Thus, on the latter time scale, the fluorescence

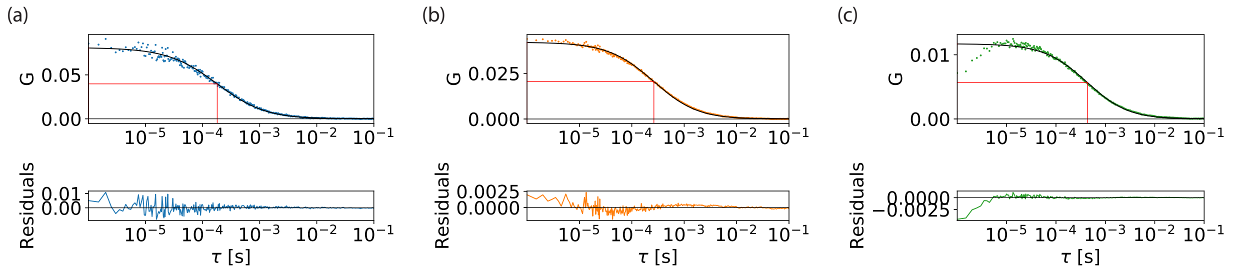

Figure S3. FCS curves for the Alexa488 sample measured with a BCD SPAD detector. auto-correlation curves and fits are shown for (a) I_{12}, (b) I_{sum3x3}, and (c) I_{sum5x5}. The decline in G for short lag times in (c) is an artefact caused by hot pixels near the edge of the detector. The fitted diffusion times indicated by the red lines are $181\ \mu s$, $263\ \mu s$, and $426\ \mu s$, respectively.

intensity decays quickly while the afterpulsing photons are nearly uniformly distributed. Given the single-photon timing capability of our SPAD array detector, this property can be exploited to remove the afterpulsing contribution from the measured signal. In the main text, we show three techniques: cropping the ACF, including an afterpulsing term in the ACF model, and calculating cross-correlations.

Crosstalk

When a photon hits a pixel of the SPAD array detector and triggers an avalanche, the carriers flowing inside the diode may cause the emission of secondary photons[4]. These secondary photons can be detected by neighbouring pixels. This phenomenon is called optical crosstalk and degrades the signal-to-noise ratio (SNR). However, for the SPAD array detector used here, the crosstalk probability is less than 1.5% for first orthogonal neighbours and less than $0.2\ \%$ for first diagonal neighbours. In addition, the time difference between the detection of a genuine fluorescence photon and the detection of a crosstalk photon is less than $1\ ns$, *i.e.,* much shorter than the $500\ ns$ bin time of the FCS measurements. As a result, the cross-correlation curves of Figure S2 (d) do not show any indication of crosstalk.

Supplementary Note 3: Validation of the setup

We validated our data acquisition and analysis platform by performing FCS on the Alexa 488 sample. The SPAD array detector was replaced with a single-element SPAD (Micro Photon Devices, Bolzano, Italy), which was simultaneously connected to one of the 25 channels of our data acquisition platform and a Becker and Hickl time tagging card (SPC-830, Becker and Hickl GmbH, Berlin, Germany). In Figure S4 (a-b) the auto-correlation curve as calculated by the Becker and Hickl software from the photon arrival times is compared with the FCS curve calculated in Python obtained from the binned data as measured by our platform. The results are in very good agreement. Minor differences can be explained by the different protocols for acquiring the data, e.g. time tagging vs. binning, and correspondingly, the different algorithms for calculating the correlation function.

Figure S4 (c) shows a comparison of the auto-correlation curve measured by the single-element SPAD and by summing the signal of all pixels of the SPAD array detector. Both FCS measurements were performed consecutively on the same Alexa 488 sample and under the same experimental conditions, *i.e.,* using the same NI data acquisition platform, the same laser power (8 μW average power) and the same measurement duration (60 s). For visualization purposes, both curves have been normalized to 1. There is good agreement between the two ACFs. The SPAD array detector shows a somewhat lower SNR, and consequently a higher χ^2 value, which can be attributed to the lower photon detection efficiency, mainly caused by the dead area in between the pixels and the fact that every individual pixel adds noise to the overall signal. However, as we show in this paper, the sensitivity and the SNR of the SPAD array detector is high enough to do FCS measurements, even on dim and quickly moving particles. The longer diffusion time measured with SPAD-array-detector compared to the single-element SPAD can be attributed to the larger field-of-view of the array detector, about 1.5 A.U. compared to 1.1 A.U. for the single-element SPAD.

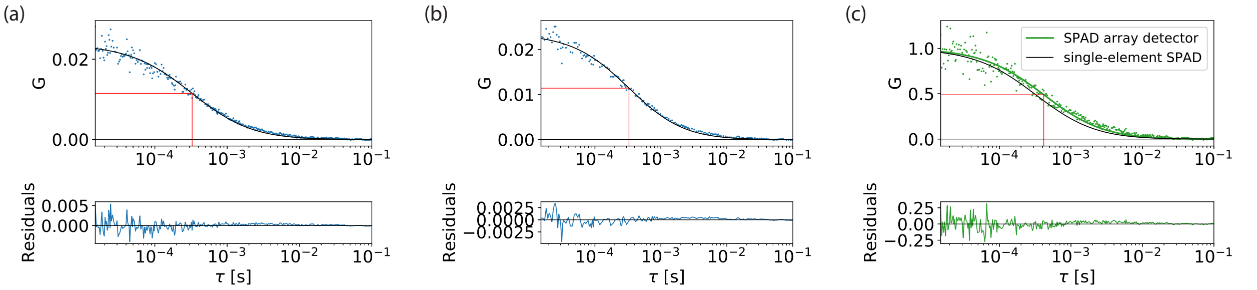

Figure S4. FCS measurement on the Alexa 488 sample with a single-element SPAD simultaneously connected to (a) our NI platform and (b) a Becker and Hickl time tagging platform for reference. The fitted diffusion times are $328\ \mu s$ and $330\ \mu s$, respectively. (c) Comparison between conventional FCS with a point-detector (i.e., the plot from panel (a)) and SPAD-array-FCS with the photon counts over all pixels summed. Both curves are normalized to 1 for direct comparison. The fitted diffusion time for the SPAD-array-detector is $421\ \mu s$ (shown in red). The χ^2 values for the normalized curves are 0.14 for the SPAD-array-detector and 0.087 for the single-element SPAD.

Supplementary Note 4: Lateral shifts in the field-of-view in pair-correlation analysis

In real space the distance between two neighbouring pixels is $75\ \mu m$. The magnification of the system is 500, so in the sample plane the pixel-pixel distance is $150\ nm$. Given that the overall detection volume (or system PSF) is the product of the excitation and the emission PSFs, and assuming a reassignment factor of 1/2 [ref. 7], the shifts in the field-of-view of two neighbouring pixels is $75\ nm$.

Table S1. Overview of the different data analysis methods.

Method	Working principle	Result	Limitations and solutions
Spot-variation FCS	Generate new intensity traces recorded with different virtual pinhole sizes by summing the signal from different pixels. The autocorrelation of each new trace is calculated and the diffusion time is fitted as a function of the lateral PSF size.	$\tau(\omega_0^2)$ is a linear function. For free diffusion, the curve passes through the origin, and the diffusion coefficient can be derived from the slope. For anomalous diffusion, the intercept value $\tau(0)$ will be positive for diffusion in microdomains and negative for diffusion hindered by a meshwork[1].	· The most commonly used fit model for G assumes a Gaussian PSF. This is a good approximation for small pinhole sizes. However, if the field-of-view of the detector is more than 1 A.U., the PSF for pixels far away from the central pixel will significantly deviate from a Gaussian function[2]. Instead one can use a more complex numerical model, see e.g.[3]. Alternatively, the zoom of the optical system can be increased to reduce the field-of-view of the array detector to not more than 1 A.U.<br>· The afterpulsing effect is still present when summing the signal from different pixels. This can be filtered out by cropping G, using lifetime information, or by calibrating the afterpulsing component.
Pair-correlation FCS / Two-focus FCS	The intensity trace from the central pixel is taken as a reference, and all 24 cross-correlations between the central pixel and the other pixels are calculated. All correlation curves that correspond to the same inter-pixel distance are averaged, resulting in 5 final cross-correlation curves. These can be fitted simultaneously, *i.e.*, in a global fit, or separately, with the two-focus FCS model.	The diffusion coefficient can be extracted from the fits. The absence of afterpulsing in the cross-correlations makes this method suitable for measuring fast diffusion. The well-known distance between the foci yields a higher precision compared to conventional fluorescence cross-correlation spectrosocpy.	· By averaging over all pixel-pairs with the same inter-pixel distance, any anisotropy in G remains undetected. E.g. the cross-correlations $G_{12,11}$ and $G_{12,13}$ are different when there is active transport in the horizontal direction. The loss of directionality hinders the study of anomalous diffusion.<br>· The Gaussian model that is assumed is not valid for large inter-pixel distances, leading to poor fit results.<br>· The information from only 24 cross-correlations is used. This number can be doubled by calculating the cross-correlations in both directions, e.g. both $G_{12,13}$ and $G_{13,12}$. However, this is still a small fraction of the total amount of information (25x25 = 625 correlations) contained in the data.
spatio-temporal image correlation spectroscopy with mean squared displacement analysis	Each 5x5 frame is treated like an image and the spatio-temporal correlations are calculated in similar way as in STICS: $G(\xi,\psi,\tau)$ contains the correlation value averaged in space and time over all pairs $(I(x,y,t),\ I(x-\xi,y-\psi,t-\tau))$. Note that $G(\xi,\psi,\tau)$ is not necessarily equal to $G(-\xi,-\psi,\tau)$. The 9x9 pixel correlation images $G_\tau(\xi,\psi)$ are fitted with a 2D Gaussian function and the variance $\sigma^2(\tau)$ and the peak location $(x_0(\tau),y_0(\tau))$ are analyzed.	Assuming equally sized Gaussian PSFs for all pixels, there is a linear relationship between σ^2 and τ: $\sigma^2 = 2D\tau + \omega_0^2/2$. The diffusion coefficient can thus be derived from the slope of σ^2 without needing a-priori knowledge on the beam waist. In addition, if different values are found for σ^2 in the ξ and ψ direction, this indicates hindered Brownian motion (Gratton). The speed and direction of active transport (e.g. superimposed on Brownian motion) can be derived from the peak location. Knowing the geometry of the detector and the magnification of the system, this analysis method is completely calibration-free.	· All PSFs are approximated by a Gaussian function with the same width ω_0. This approximation is less accurate for pixels near the edge of the detector, see *Spot-variation FCS*. This approximation is worse in STICS analysis than in pair-correlation analysis, since STICS also analyses pair-correlations between pixels located on opposite sides of the detector, thus with higher inter-pixel distances.<br>· $G(0,0,\tau)$ contains the afterpulsing component for small τ values. This effect can be filtered out by using a weighted fit with low (or zero) weights for $G(0,0,\tau<\tau_{AP})$, with τ_{AP} the maximum lag time between a photon signal and the afterpulsing signal.

References

1. Wawrezinieck, L., Rigneault, H., Marguet, D. & Lenne, P. F. Fluorescence Correlation Spectroscopy Diffusion Laws to Probe the Submicron Cell Membrane Organization. *Biophys. J.* **89,** 4029–4042 (2005).
2. Sheppard, C. J. R. *et al.* Pixel Reassignment in Image Scanning Microscopy: A Re-Evaluation. *J. Opt. Soc. Am. A* **37,** 154–162 (2020).
3. Dertinger, T. *et al.* Two-Focus Fluorescence Correlation Spectroscopy: A New Tool for Accurate and Absolute Diffusion Measurements. *ChemPhysChem* **8,** 433–443 (2007).
4. Buttafava, M. *et al.* Spad-Based Asynchronous-Readout Array Detectors for Image-Scanning Microscopy. *Optica* **7,** 755–765 (2020).
5. Colyer, R. A. *et al.* High-Throughput Fcs Using an Lcos Spatial Light Modulator and an 8 X 1 Spad Array. *Biomed. Opt. Express* **1,** 1408–1431 (2010).
6. Enderlein, J. & Gregor, I. Using Fluorescence Lifetime for Discriminating Detector Afterpulsing in Fluorescence-Correlation Spectroscopy. *Rev. Sci. Instrum.* **76,** 033102 1–5 (2005).
7. Sheppard, C. J. R., Castello, M., Tortarolo, G., Vicidomini, G. & Diaspro, A. Image Formation in Image Scanning Microscopy, Including the Case of Two-Photon Excitation. *J. Opt. Soc. Am. A* **34,** 1339–1350 (2017).
